# Laparoscopic median arcuate ligament section for median arcuate ligament compression syndrome initially detected as splenic infarction: a case report

**DOI:** 10.1186/s40792-024-01817-w

**Published:** 2024-02-15

**Authors:** Toru Takagi, Yoshifumi Morita, Akio Matsumoto, Shinya Ida, Ryuta Muraki, Ryo Kitajima, Satoru Furuhashi, Makoto Takeda, Hirotoshi Kikuchi, Yoshihiro Hiramatsu, Hiroya Takeuchi

**Affiliations:** 1 Department of Surgery, Hamamatsu University School of Medicine, 1-20-1 Handayama, Higashi-ku, Hamamatsu, 431-3192 Japan; 2 Department of Perioperative Functioning Care & Support, Hamamatsu University School of Medicine, Hamamatsu, 1-20-1 Handayama, Chuo-ku, 431-3192 Japan; 3 Division of Surgical Care, Morimachi, Hamamatsu University School of Medicine, Hamamatsu, 1-20-1 Handayama, Chuo-ku, 431-3192 Japan

**Keywords:** Median arcuate ligament compression syndrome, MALS, Splenic infarction

## Abstract

**Background:**

Median arcuate ligament compression syndrome (MALS) causes upper abdominal pain and at times hemodynamic abnormalities in the pancreaticoduodenal region.

Herein, we present a case of a 70 year-old man, initially diagnosed with splenic infarction and was successfully treated laparoscopically.

**Case presentation:**

A 70-year-old man with abdominal pain admitted to our hospital. Abdominal-enhanced computed tomography revealed a poorly contrasted area in the spleen and stenosis at the root of the celiac artery. Arterial dilatation was observed around the pancreaticoduodenal arcade, however, no obvious aneurysm formation or arterial dissection was observed. Abdominal-enhanced magnetic resonance imaging indicated the disappearance of the flow void at the root of the celiac artery. The patient had no history of atrial fibrillation and was diagnosed with splenic infarction due to median arcuate ligament compression syndrome. We performed a laparoscopic median arcuate ligament section with five ports. Intraoperative ultrasonography showed a retrograde blood flow in the common hepatic artery and the celiac artery. After releasing the compression, the antegrade blood flow from the celiac artery to the splenic artery, and the common hepatic artery were visualized using intraoperative ultrasonography. The postoperative course of the patient was uneventful, and he was discharged on postoperative day 9. Postoperative computed tomography a month after surgery revealed no residual stenosis of the celiac artery or dilation of the pancreaticoduodenal arcade. Furthermore, the poorly contrasted area of the spleen improved.

**Conclusions:**

Reports indicate that hemodynamic changes in the abdominal visceral arteries due to median arcuate ligament compression are related to the formation of pancreaticoduodenal aneurysms. In this case, median arcuate ligament compression syndrome caused splenic infarction by reducing blood flow to the splenic artery.

## Background

The median arcuate ligament (MAL) is the union of the left and right legs of the diaphragm at the anterior of the vertebral body. Median arcuate ligament compression syndrome (MALS) is rare and is commonly observed in women with a thin body habitus in the 3rd to 5th decade of life [1]. The syndrome consists of postprandial symptoms including pain, nausea, vomiting, diarrhea, and unexplained weight loss [2, 3]. MALS may causes hemodynamic abnormalities including aneurysm rupture around the pancreaticoduodenal region [4–7].

MALS is rarely detected in splenic infarction [8, 9]. Herein we report a case of MALS presenting as splenic infarction that was successfully treated with laparoscopic MAL section a minimally invasive surgery.

## Case presentation

**Fig. 1:**
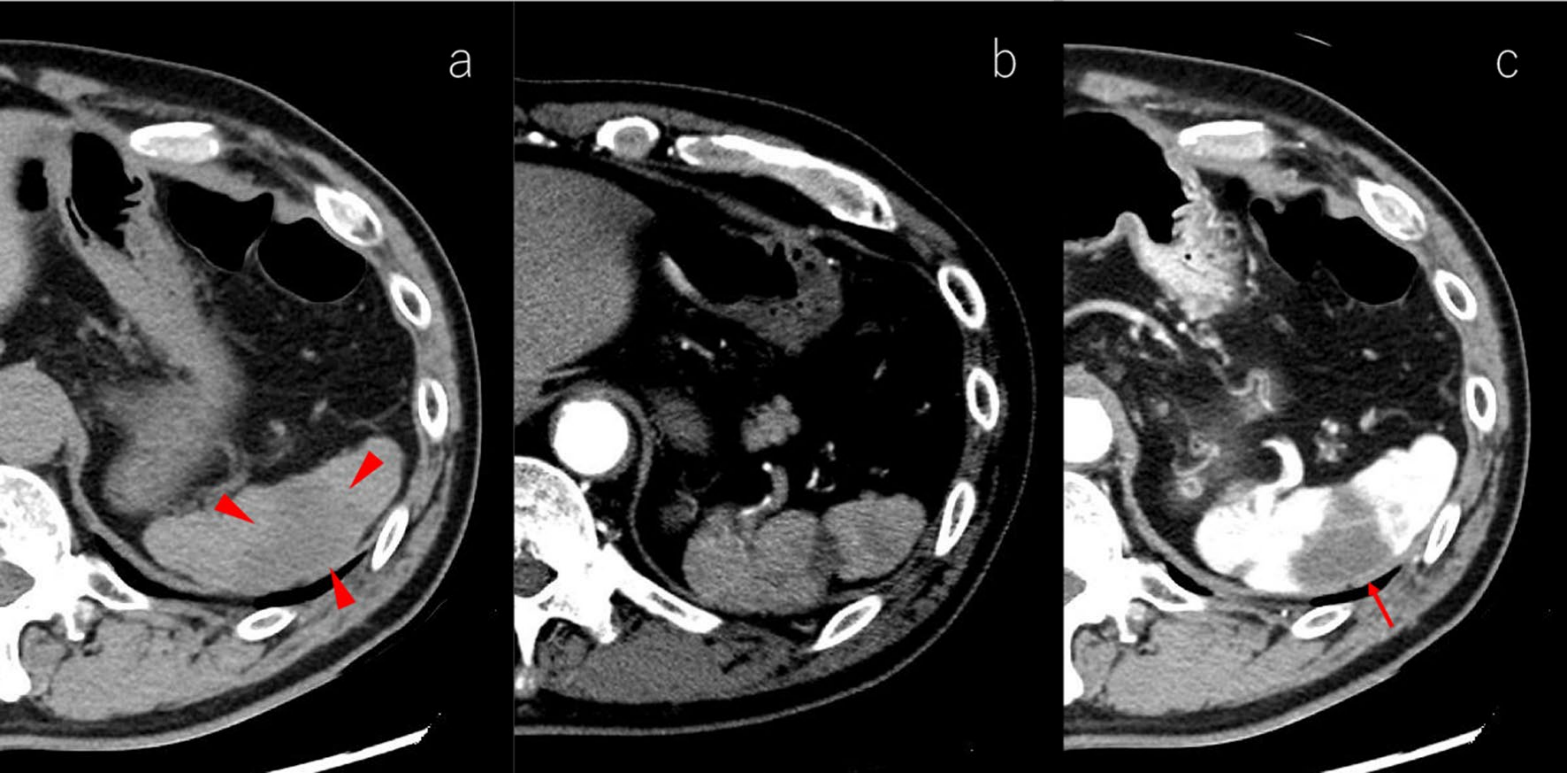
Plane and Enhanced CT findings (axial). **a** Plane CT shows a slightly low-density area (red arrowheads) compared with the surrounding area in the spleen. **b**, **c** Arterial and venous phases of enhanced CT indicate a poorly contrasted area in the spleen (red arrow)

**Fig. 2:**
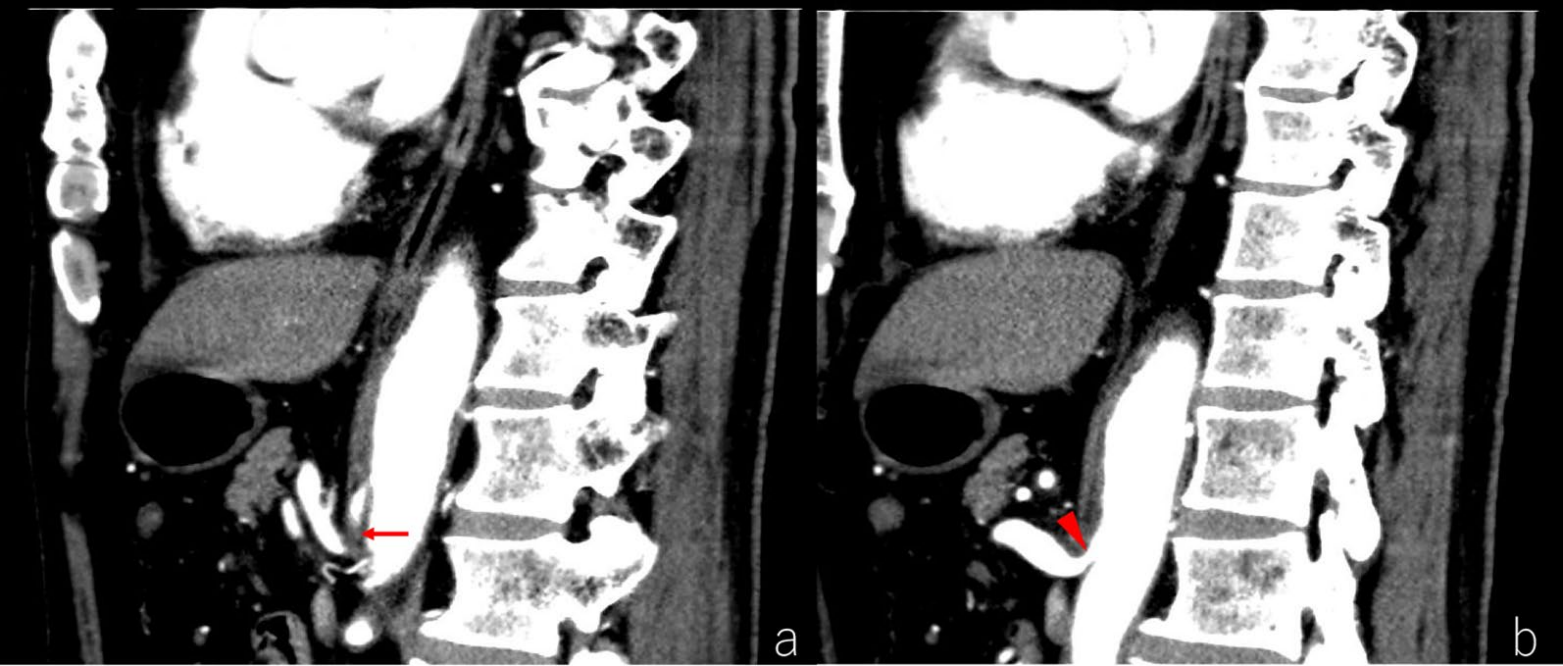
Enhanced CT findings (sagittal). **a** The arterial phase of enhanced CT reveals the almost occluded celiac arterial root (red arrow) and post-stenotic dilatation. **b** Arterial-phase enhanced CT displays mild stenosis at the root of the superior mesenteric artery (red arrowhead)

**Fig. 3:**
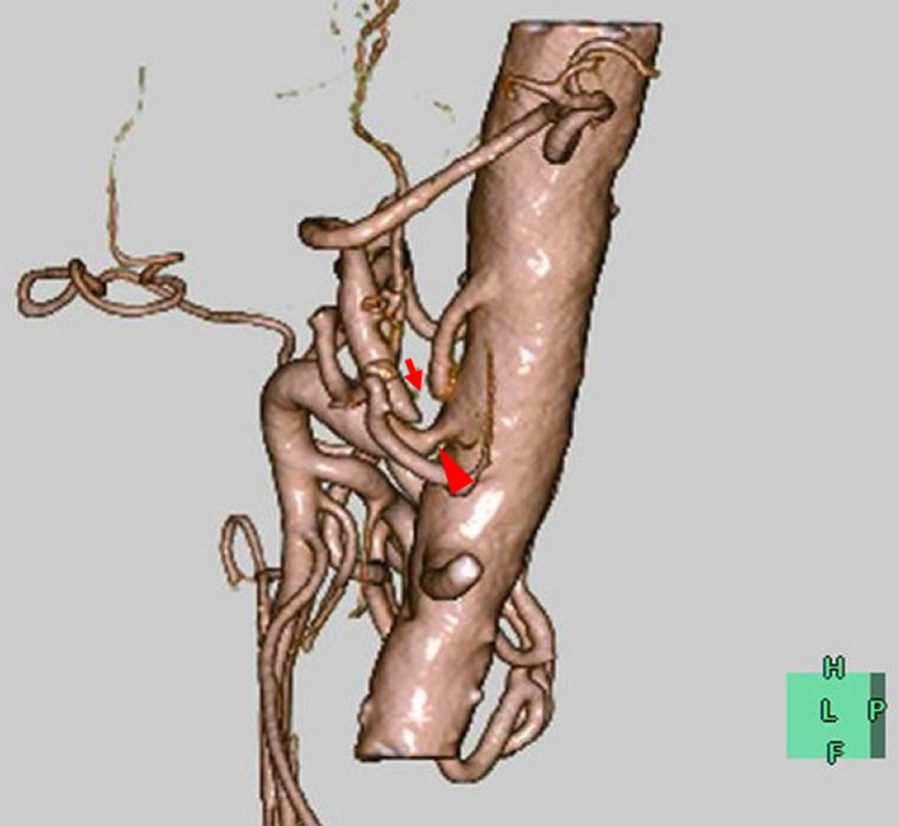
Three-dimensional reconstruction image of enhanced CT. Three-dimensional reconstructed image indicates an almost occluded celiac arterial root (red arrow) and mild stenosis at the root of the superior mesenteric artery (red arrowhead)

A 70-year-old man with abdominal pain was admitted to our hospital. Enhanced abdominal computed tomography (CT) revealed a poorly contrasted area in the spleen and stenosis at the root of the celiac artery (CA) (Fig. 1). Sagittal image and 3-D reconstruction image of enhanced CT indicated the almost occluded celiac arterial root, and post-stenotic dilatation (Figs. 2, 3). Arterial dilatation was observed around the pancreaticoduodenal arcade. However, no obvious aneurysm formation or arterial dissection was observed. Abdominal-enhanced magnetic resonance imaging (MRI) depicted the disappearance of the flow void at the root of the CA. The patient had no history of atrial fibrillation but was diagnosed with splenic infarction due to MALS.

**Fig. 4:**
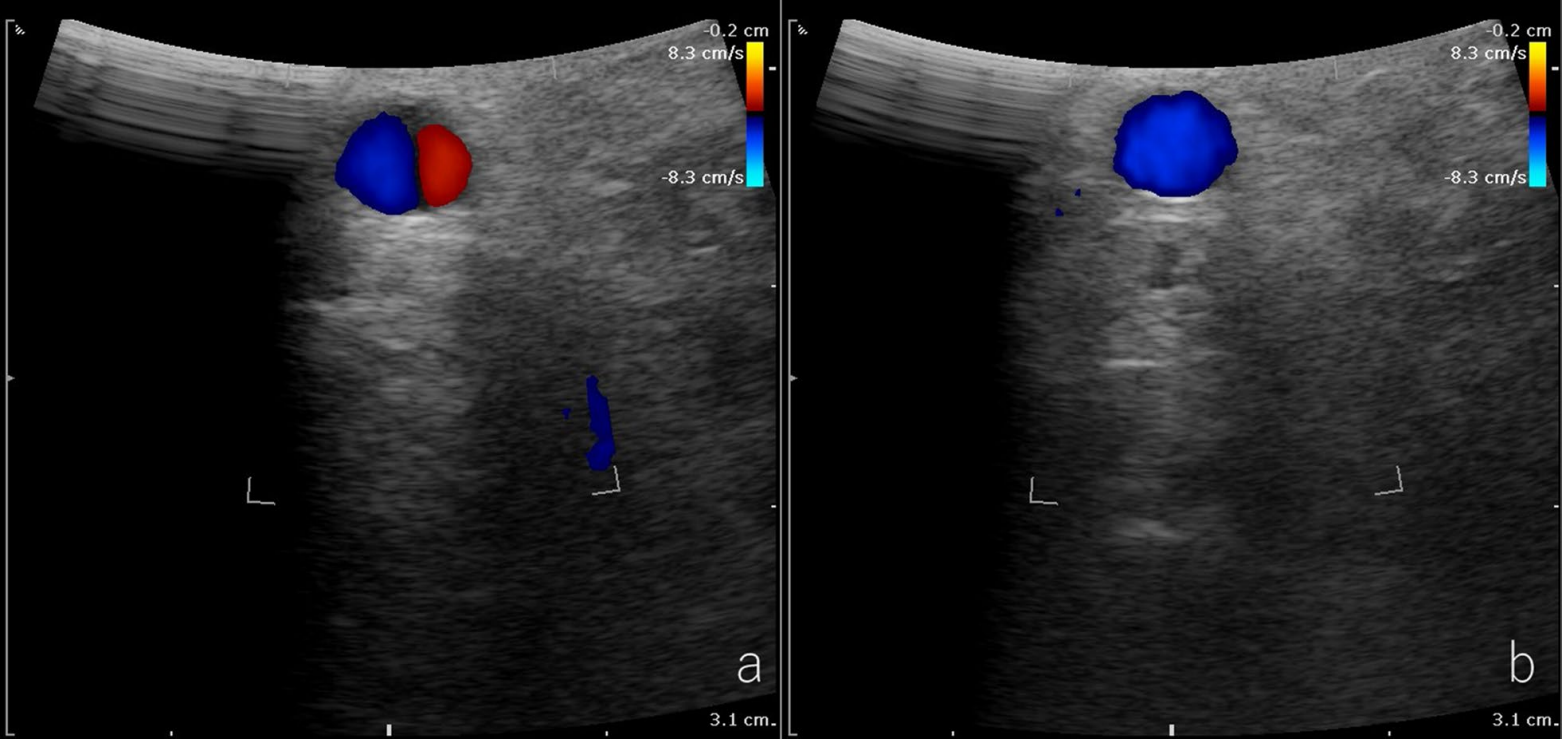
Intraoperative ultrasonography of CHA. a: Before sectioning the median arcuate ligament, retrograde blood flow in the common hepatic artery (CHA) is observed. **b** After releasing the compression, antegrade blood flow in the CHA is depicted

**Fig. 5:**
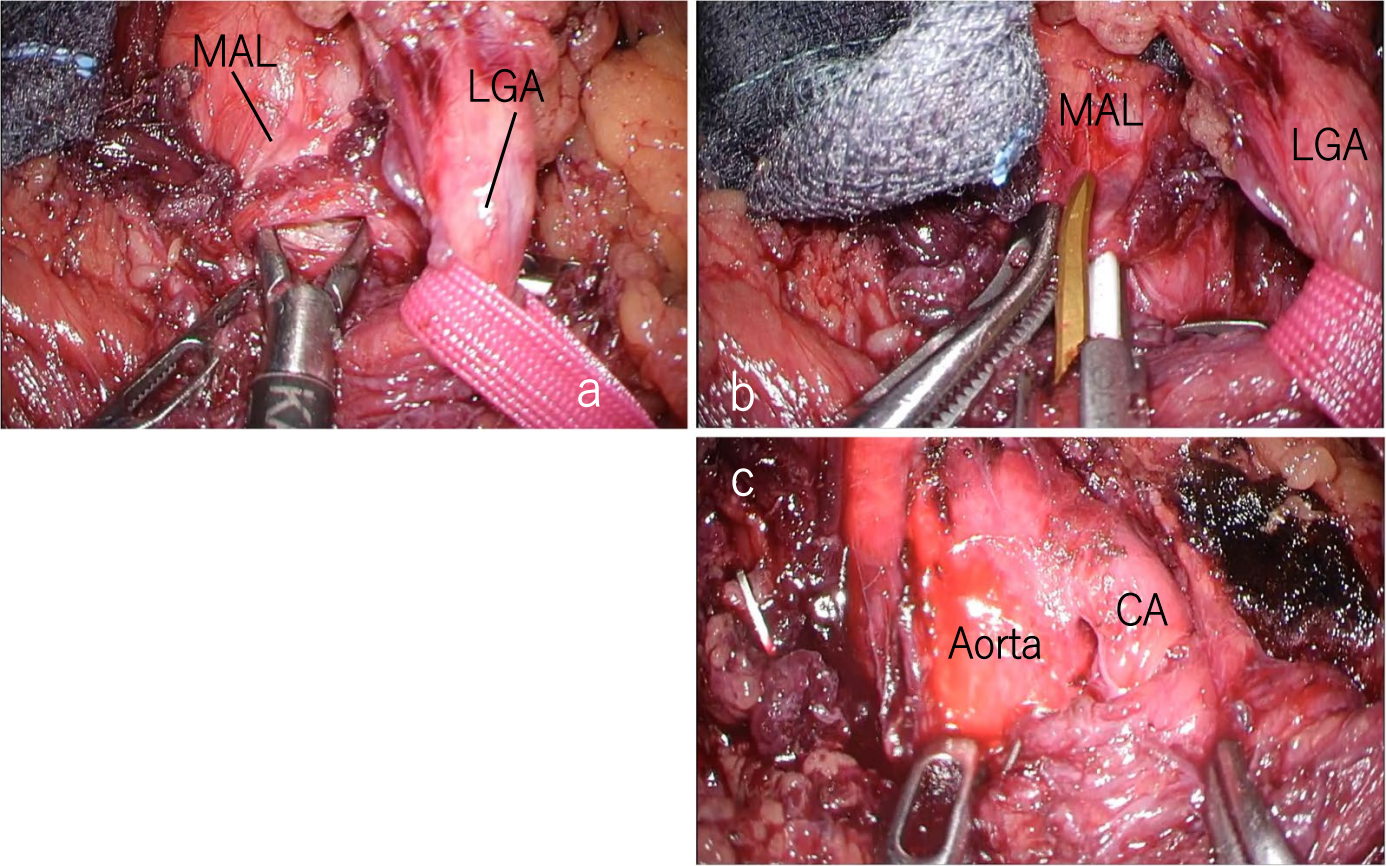
Intraoperative findings. **a** After exposure of the diaphragmatic crura and left gastric artery, the muscular fiber covers the abdominal aorta and around the celiac axis. **b**, **c** The compressing ligaments are divided until the three-quarters circumferential aortoceliac artery bifurcation is confirmed

**Fig. 6:**
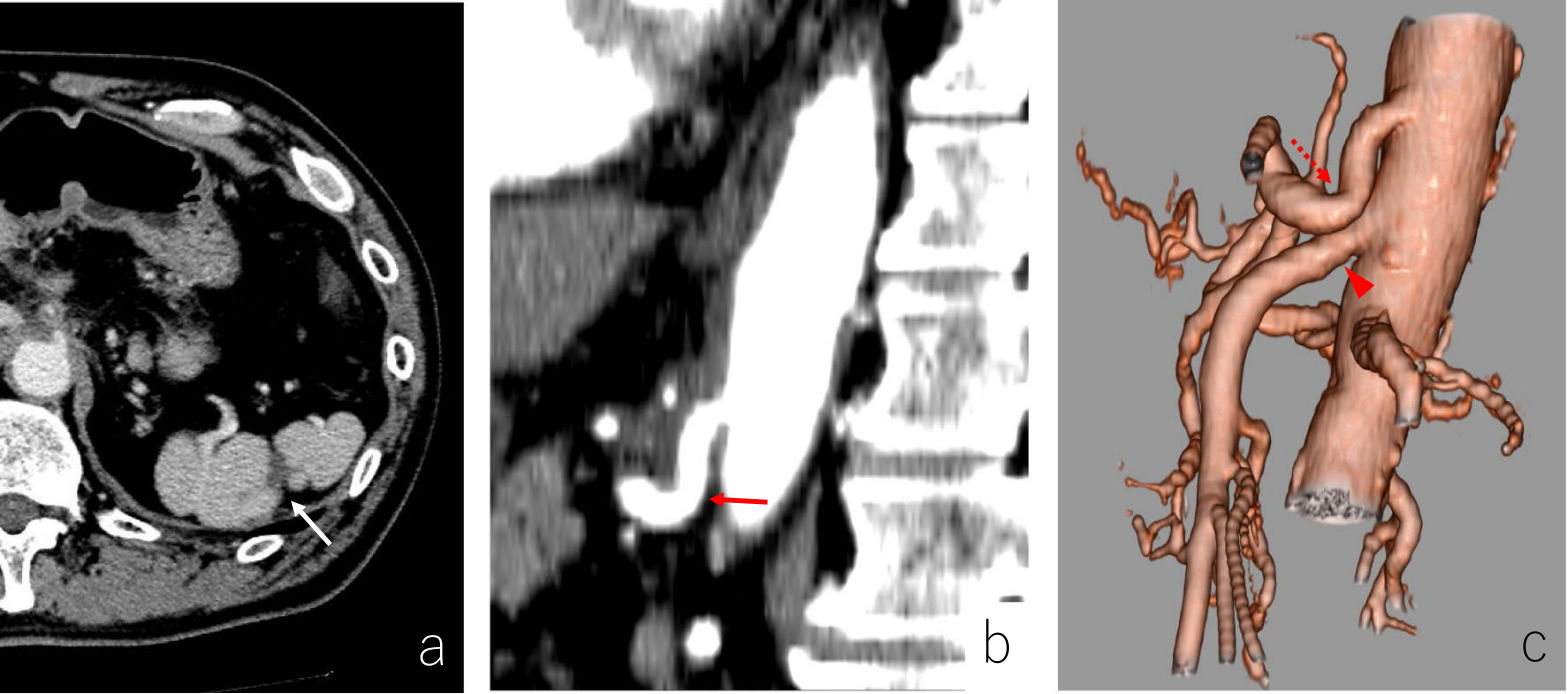
**Pos**toperative enhanced CT. **a** A month after surgery, enhanced CT reveals that the poorly perfused region of the spleen is markedly reduced (white arrow). **b** Sagittal image of enhanced CT shows improvement of stenosis of the celiac arterial root (red arrow). **c** Three-dimensional reconstructed image indicates improvement of stenosis at the root of the celiac artery (dotted red arrow) and the superior mesenteric artery (red arrowhead)

We performed laparoscopic MAL section as an elective surgery. Under general anesthesia, the first port for the videoscope was inserted at the umbilicus using the open technique. The other four operating ports were inserted after establishing pneumoperitoneum. After dividing the lesser omentum, the stomach was retracted using a Penrose drain. Furthermore, the lateral segment of the liver was ventrally retracted using Nathanson Hook Liver Retractors with Silicone Disk. The left gastric artery was looped with vessel tape and extracorporeally retracted. These retractions freed the hands of the surgeon and increased safety in probable emergency situations. Laparoscopic intraoperative ultrasonography (IOUS) indicated retrograde blood flow in the common hepatic artery (CHA) and the CA (Fig. 4a). After exposing the diaphragmatic crura and left gastric artery, muscular fibers covered the abdominal aorta and the area around the celiac axis (Fig. 5a). Further cranial dissection revealed that the MAL compressed the CA. These compressing ligaments were divided until a three-quarters circumferential aortoceliac artery bifurcation was observed (Fig. 5b, c). Improvement in the arterial flow was confirmed during surgery using laparoscopic IOUS (Fig. 4b). After releasing the compression, antegrade blood flow from the CA to the CHA and the splenic artery (SPA) was depicted using IOUS. The operative time was 226 min and the intraoperative blood loss was 15 mL. The patient was discharged nine days after surgery without complications. Enhanced CT performed one month after surgery revealed no residual stenosis of the CA, no dilation of the pancreaticoduodenal arcade, and a marked reduction in the poorly perfused region of the spleen (Fig. 6).

## Discussion

MALS is often detected by close examination of nonspecific gastrointestinal symptoms such as abdominal pain. Here, MALS was diagnosed following splenic infarction due to decreased blood flow to the spleen and was successfully treated by laparoscopic surgery.

MALS generally causes postprandial abdominal pain, weight loss, bloating, nausea, and vomiting. Several reports have suggested that duplex ultrasonography (US) and abdominal CT are useful for the diagnosis [10, 11]. Horton et al. reported hooked appearance as a characteristic finding on CT [12]; however, the celiac artery was almost occluded in this case and no such appearance was observed.

MALS can cause hemodynamic abnormalities in the pancreaticoduodenal region. It has been reported that 68–74% of patients with pancreaticoduodenal aneurysms have stenosis of the origin of the CA, and 80% of patients with stenosis of the origin of the CA have arterial dilatation of the pancreatic head arcade [4]. We previously reported blood flow assessment using four-dimensional flow magnetic resonance imaging (4D-flow MRI) [13]. 4D-flow MRI is useful for evaluating the hemodynamics of patients with MALS preoperatively and postoperatively.

The typical treatments are transcatheter endovascular therapy or surgery. Traditionally, decompression of the CA has been achieved through an open approach, in which a chevron or upper midline laparotomy permits the identification of the MAL and CA. Subsequently, the compressive band is divided along with division and wide excision of the celiac ganglion (ganglionectomy), and the proximal CA is completely exposed. As an assessment of treatment, IOUS objectively demonstrates a return to normal peak systolic velocities [14]. Recently, intraoperative indocyanine green (ICG) fluorescence angiography has been advocated as an effective tool for evaluating hemodynamics. ICG fluorescence angiography may intuitively determine multiple vessels in the operative field simultaneously [15]; however, objective evaluation of blood flow and velocity is challenging. In contrast, Doppler US is useful for objective evaluation, but cannot evaluate multiple vessels simultaneously.

In recent years, laparoscopic interventions are employed to decompress the CA. We previously reported that the laparoscopic ligament section is a useful treatment for MALS [16]. Since each method of evaluating the effect of the MAL section on blood flow has its own advantages and disadvantages, it is recommended that each institution make effective use of available equipment. With laparoscopic ultrasonography, measuring arterial flow velocity is possible and preferable for quantitative evaluation. A fluorescence-observable laparoscope or intraoperative radiography allows intuitive evaluation of multiple vessels simultaneously. Jiminez et al. reviewed studies reporting the outcomes of surgical interventions for MALS [17]. Overall, 85% (open group, 78%; laparoscopic group, 96%) of the patients reported immediate postoperative symptom relief. The late recurrence rates were 6.8% and 5.7% in the open surgery and the laparoscopic surgery groups respectively, owing to bleeding, 9.1% of the laparoscopic procedures were converted to open surgery. Robot-assisted MAL section and celiac ganglionectomy have recently emerged as treatment modalities. Do et al. stated that laparoscopic MAL section had significantly shorter operative times than robot-assisted MAL section, however, both are safe and effective interventions [18].

If the lumen of the celiac artery cannot be dilated by long-term compression or if there is mechanical obstruction, additional treatment such as bypass or interventional radiology (IVR) may be required. If MAL resection fails to improve arterial blood flow, endovascular balloon dilation or stent graft placement will become additional options. Intraoperative radiography, if available, should be prepared as a backup. In cases of aneurysms, a treatment strategy combining transcatheter arterial embolization, resection of the aneurysm, bypass, and MAL section should be considered, depending on the presence or absence of rupture [12]. However, IVR alone does not address extrinsic compression of the CA and has subsequently proven ineffective as an isolated treatment of MALS [19].

## Conclusion

Although splenic infarction due to decreased blood flow in the splenic artery is a rare complication in MALS, it should be kept in mind.

## Data Availability

Not applicable.

## Abbreviations

MAL: Median arcuate ligament
MALS: Median arcuate ligament compression syndrome
CT: Computed tomography
CA: Celiac artery
MRI: Magnetic resonance imaging
IOUS: Intraoperative ultrasonography
CHA: Common hepatic artery
SPA: Splenic artery
US: Ultrasonography
IVR: Interventional radiology
4-D flow MRI: Four-dimensional flow MRI
ICG: Indocyanine green
